# Metabolomic analysis and antibacterial and antioxidant activities of three species of *Artemisia* plants in Tibet

**DOI:** 10.1186/s12870-023-04219-6

**Published:** 2023-04-21

**Authors:** Xinyu Liu, Renzengwangdui, Shiyu Tang, Yiru Zhu, Meng Wang, Binqian Cao, Jinglong Wang, Baoyu Zhao, Hao Lu

**Affiliations:** 1 College of Veterinary Medicine, Northwest A&F University, Yangling, 712100 Shaanxi China; 2 Tibet Academy of Agricultural and Animal Husbandry Sciences/State Key Laboratory of Barley and Yak Germplasm Resources and Genetic Improvement, Lhasa, 850002 Tibet China

**Keywords:** *Artemisia*, Mmetabolomic analysis, Antibacterial activity, Antioxidant activity

## Abstract

**Background:**

*Artemisia* is important medicinal plants in China and are widely used in medicine, agriculture, and food. Pharmacologically active components of the plants remain to be investigated.

**Methods:**

This study sought to identify and compare the chemical constituents of three species of *Artemisia* in Tibet using a widely-targeted metabolomics approach and their antibacterial and antioxidant capacities were determined.

**Result:**

A total of 1109 metabolites within 10 categories were detected from the three species of *Artemisia*, including lipids, amino acids, nucleotides, flavonoids, terpenes, coumarins, organic acids, and phenolic acids. 732 different metabolites have been identified between *Artemisia sieversiana* and *Artemisia annua*, 751 different metabolites were identified between *Artemisia wellbyi* and *A. sieversiana*, and 768 differential metabolites were differentially detected from *A. wellbyi* and *A. annua.* Differentially identified compounds included flavonoids, phenolic acids, artemisinins and coumarin. *A. annua* contained the highest relative content of artemisinin among three *Artemisia*. The antimicrobial experiments showed that the three *Artemisia* species had strong antibiotic activities against *Bacillus subtilis*, *Escherichia coli*, *Staphylococcus aureus*, *Proteus mirabilis* and *Pseudomonas aeruginosa*. The biochemical analysis showed that the three species of *Artemisia* have strong antioxidant capacity.

**Conclusions:**

This is the first reported attempt to comparatively determine the types of the metabolites of the three widely distributed *Artemisia* species in Tibet. The information should help medicinal research and facilitate comprehensive development and utilization of *Artemisia* species in Tibet.

**Supplementary Information:**

The online version contains supplementary material available at 10.1186/s12870-023-04219-6.

## Background

*Artemisia* sp. plants belong to the *Compositae* families Anthemideae and Artemisiinae [1]. There have been estimated total of 344 species and 69 varieties of *Artemisia* in the world; Asia contains the most types with 269 species and 60 varieties [2]. There are 187 species and 46 varieties of *Artemisia* have been found throughout China [3]. *Artemisia* is tenacious and can grow at high altitude and in extremely arid areas [4, 5] which are widely distributed in Tibet and are the major species in desert steppe and grassland. Survey results showed that 57 species and 6 varieties of *Artemisia* species distributed in Tibet, accounting for a quarter of the national *Artemisia* species [6]. *A. wellbyi* has been shown to be of higher nutritional quality containing higher crude protein and crude fat content than other herbs found in Tibetan grasslands [7], and has the potential to become a supplementary grass seed for ecological restoration of grasslands in the Tibetan plateau [8]. *A. sieversiana* is mainly used for hay, a reserve feed for cattle and sheep in winter [9], and an important source of animal feed in Tibet [10]. In addition, *A. sieversiana* plants can be used as high-quality roughage after silage [11]. *A. annua* extracts added to feed can promote animal growth, improve the body's disease resistance, and improve animal production performance [12].

*Artemisia* contain a large class of medicines widely used traditionally by Tibetans. Traditional Chinese medicinal practitioners believe that this genus has antibacterial and anti-inflammatory and has wide range of health beneficial properties [13–15]. It is widely used in malaria, hepatitis, cancer, inflammation, infection and other diseases. In 2015, Tu Youyou was awarded Nobel Prize in Physiology or Medicine for her discovery of the antimalarial sesquiterpenoid artemisinin from *A. annua*. Since then, artemisinin and *Artemisia* have attracted worldwide attention. In recent years, Xiao [16] used a variety of chromatographic methods to separate and purify the compounds from the aqueous fraction of the aerial portions of *A. annua*, and identified 15 compounds based on the physicochemical properties and NMR spectral data. Wang [17] et al. studied the chemical constituents of the whole plant of *A. annua* by chromatography with silica gel matrix and HPLC, and purified 17 compounds from the ethyl acetate extract from the ethanolic extract of *A. annua* and Zhong [18] et al. extracted.and isolated 6 flavonoids from *A. annua*.

*Artemisia* produces many medicinally important secondary metabolites that have antimicrobial and antioxidant activities. External application of artesunate can inhibit *S. aureus*, *D. Bacillus*, *B. subtilis*, *P. aeruginosa*. Three kinds of extracts of *A. annua* (petroleum ether extract I; chloroform extract II; ethanol extract III) have antifungal effects; the antifungal activity of extract III is close to that of clinical routine antifungal drug [19]. *Artemisia* essential oils have strong antibacterial effects on* S. aureus*, *S. epidermidis*, *E. coli*, and *Streptococcus*, and have strong antioxidant effects [20].

While the metabolites from *A. annua* have been well characterized, the metabolites from other *Artemisia* sp. and particularly those from Tibet have not been thoroughly identified. Presently, the types of metabolites of *Artemisia* plants and the differences in metabolites among these plants are not clear. In addition, studies on the metabolites of the *Artemisia* genus have been limited, with low sensitivity, and relatively poor qualitative and quantitative accuracy [21–23]. Widely targeted metabolomics integrates the advantages of untargeted metabolites and targeted metabolite detection techniques to achieve high throughput, high sensitivity and broad coverage. We used metabolomic analysis to identify the metabolites from *A. sieversiana, A. wellbyi,* and *A. annua* and elucidate the differential metabolite species. The antibacterial and antioxidant capabilities were also evaluated on the three *Artemisia* species from Tibet. This study will provide new evidence for the potential medicinal use of the three Tibetan *Artemisia* species and lay the foundation for further exploration of the active constituents, their metabolic pathways, and pharmacological mechanisms of action.

## Results

### Qualitative and quantitative analysis of the metabolites

**Fig. 1:**
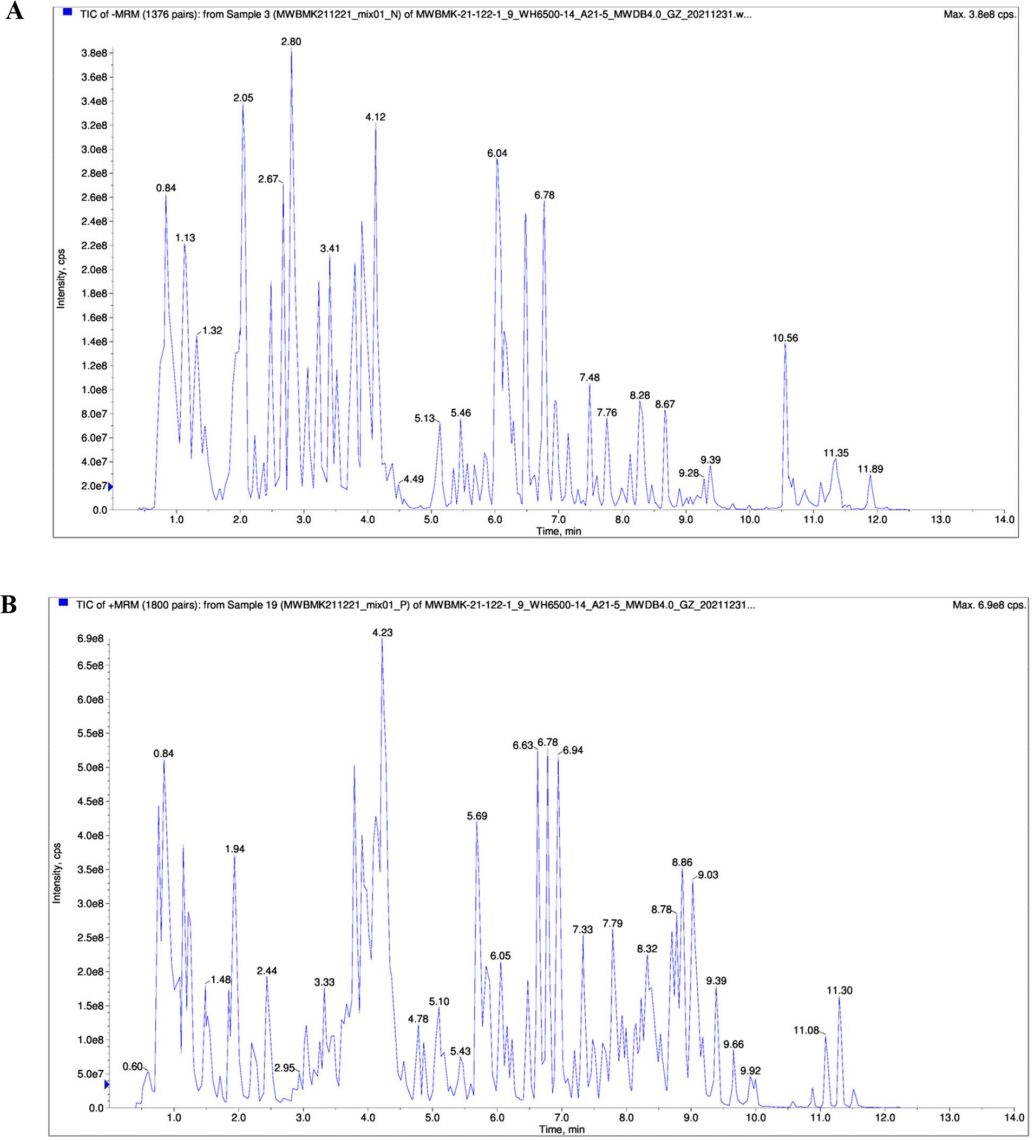
Total ion chromatograms of the metabolite analysis. **A** QC_MS_TIC-N. **B** QC_MS_TIC-P

The primary metabolites and secondary metabolites in the samples were identified by UPLC-MS. 1109 metabolites were identified from 3 species of *Artemisia*, including 79 amino acids and their derivatives, 73 nucleotides and their derivatives, 101 organic acids, 155 lipids, and 168 phenolic acids, 227 flavonoids, 40 lignans and coumarin, 86 alkaloids, 56 terpenes, and 124 others (Supplementary 1). Metabolic profiles differed by *Artemisia* species. Total ion chromatograms of the metabolite analysis were shown in Fig. 1.

### Sample quality control and statistical analysis

**Fig. 2:**
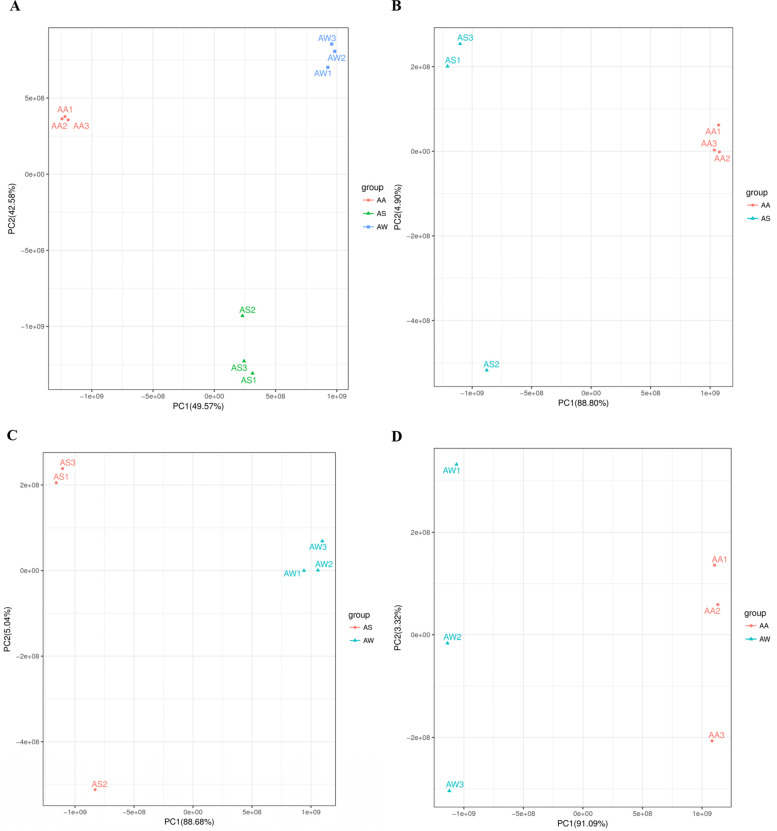
Principal component analysis. **A** All samples principal component analysis. **B** AS vs AA principal component analysis (PCA). **C** AS vs AW principal component analysis (PCA). **D** AW vs AA principal component analysis (PCA)

The results showed that the contribution rate of principal component 1 (PC1) was 49.57%, and PC2 was 42.58%, and the three groups of samples were separated in the two-dimensional diagram (Fig. 2). The differences in metabolites between the three *Artemisia* sp. are shown in the PCA results.

**Fig. 3:**
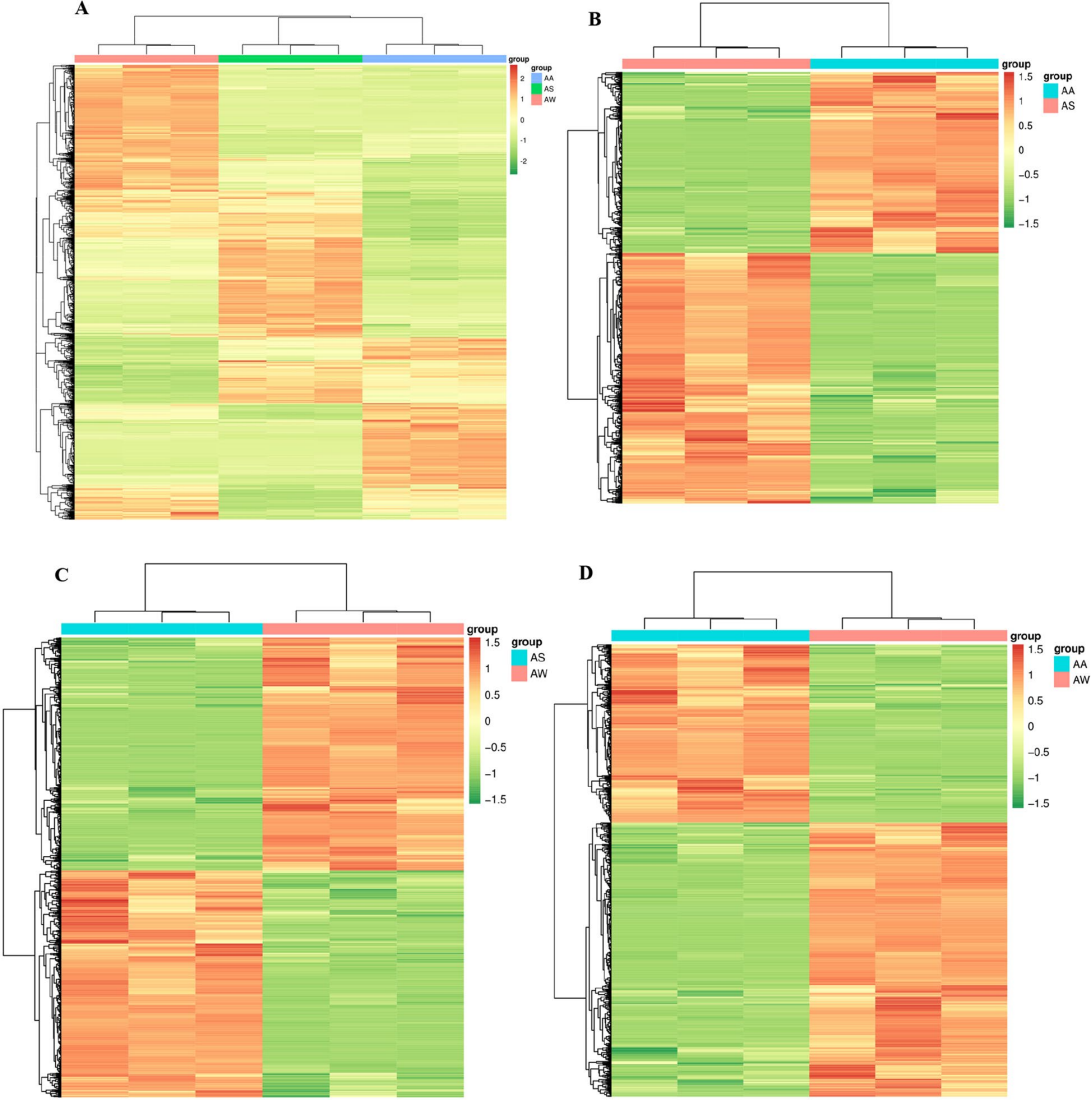
Cluster Analysis of Differential Metabolites. **A** All samples heatmap. **B** AS vs AA heatmap. **C** AS vs AW heatmap. **D** AW vs AA heatmap

Differences in accumulation patterns of metabolites from the three *Artemisia* sp. were analyzed by clustering heatmaps (Fig. 3). The heat map analysis showed the differences in substances within the plants that were grouped into 4 clusters. The metabolites in cluster 1 were the highest in AW group, medium in AS group, and AA group. Metabolites in cluster 2 were highest in AS group, moderately present in AW group, and lowest in AA group. The different biological replicates also found to be clustered together, both cluster analysis and PCA showed that metabolites were significant different in the three *Artemisia* sp.

### OPLS-DA analysis of the differential grouping

**Fig. 4:**
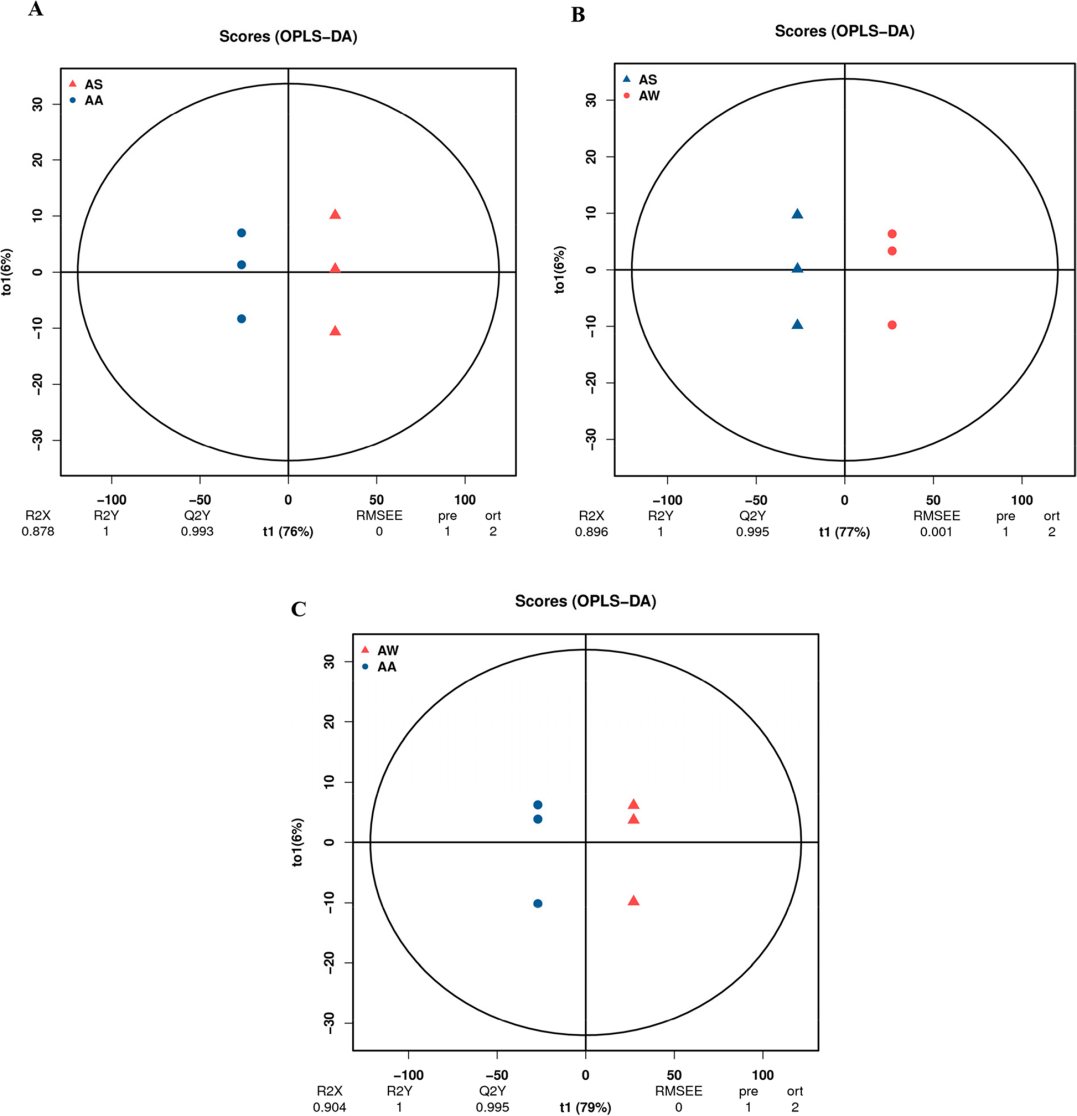
OPLS-DA score plot. **A** AS vs AA OPLS-DA. **B** AS vs AW OPLS-DA. **C** AS vs AW OPLS-DA

OPLS-DA was used to analyze the AW, AS, and AA groups in pairs to generate a score map. All the Q2 of the comparison groups were all higher than 0.9, indicating that the constructed model was suitable. According to the OPLS-DA score plot significant separation occurred in the different comparison groups. As shown in Fig. 4, the OPLS-DA model produced two principal components and the contribution rate of PC1 is 76%, and the contribution rate of PC2 is 6%. The difference between the two groups of samples is highly significant. Among the evaluation parameters of the OPLS-DA model, the indicators R^2^X = 0.878, R^2^Y = 1, Q^2^Y = 0.993 were all greater than 0.5 and Q^2^Y > 0.9, suggesting that the OPLS-DA model was correctly constructed, the prediction was reliable, and the differential metabolites could be screened according to the VIP value analysis.

### Differential metabolite screening

**Fig. 5:**
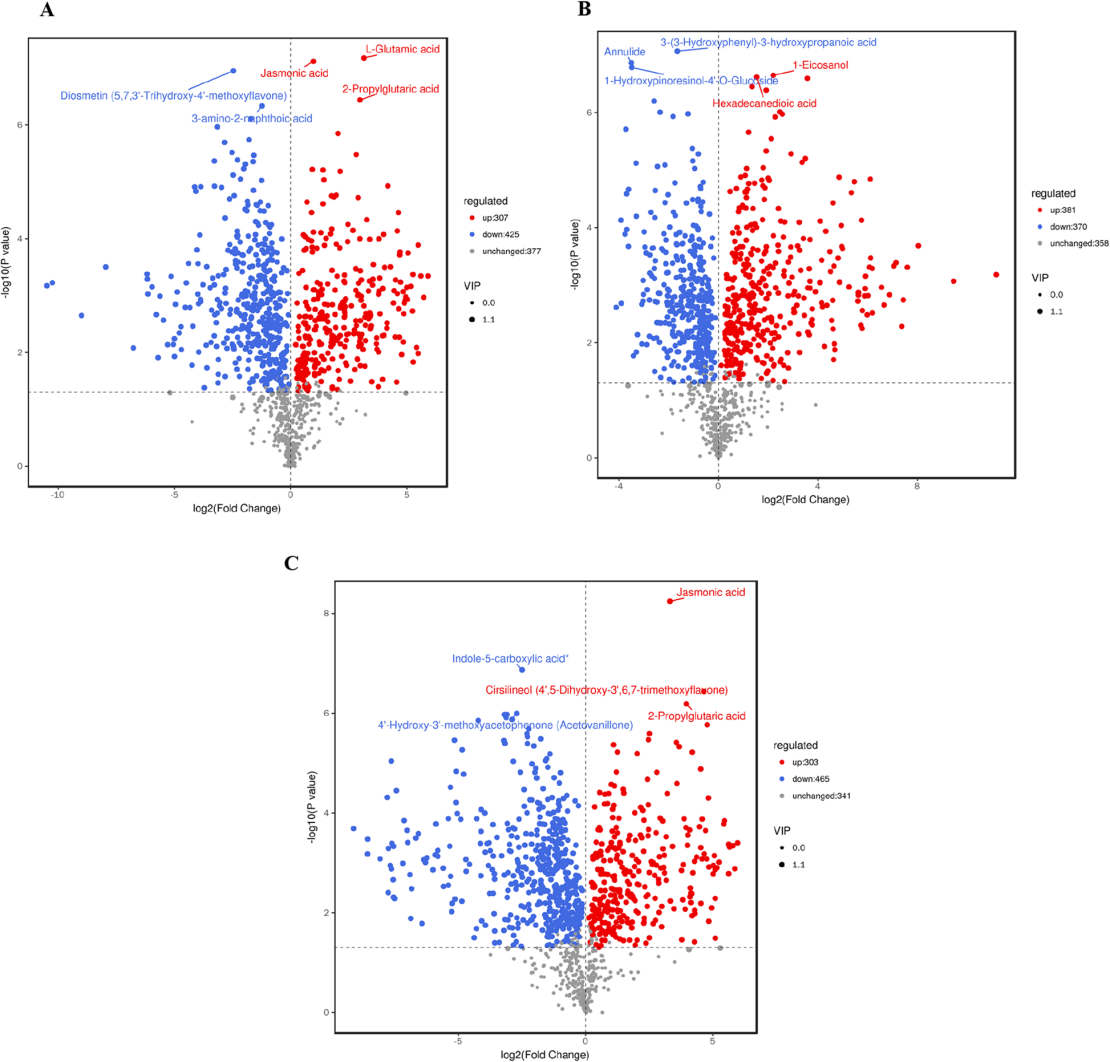
Volcano plots of the differential metabolites. **A** AS vs AA Volcano plot. **B** AS vs AW Volcano plot. **C** AS vs AW Volcano plot

**Fig. 6:**
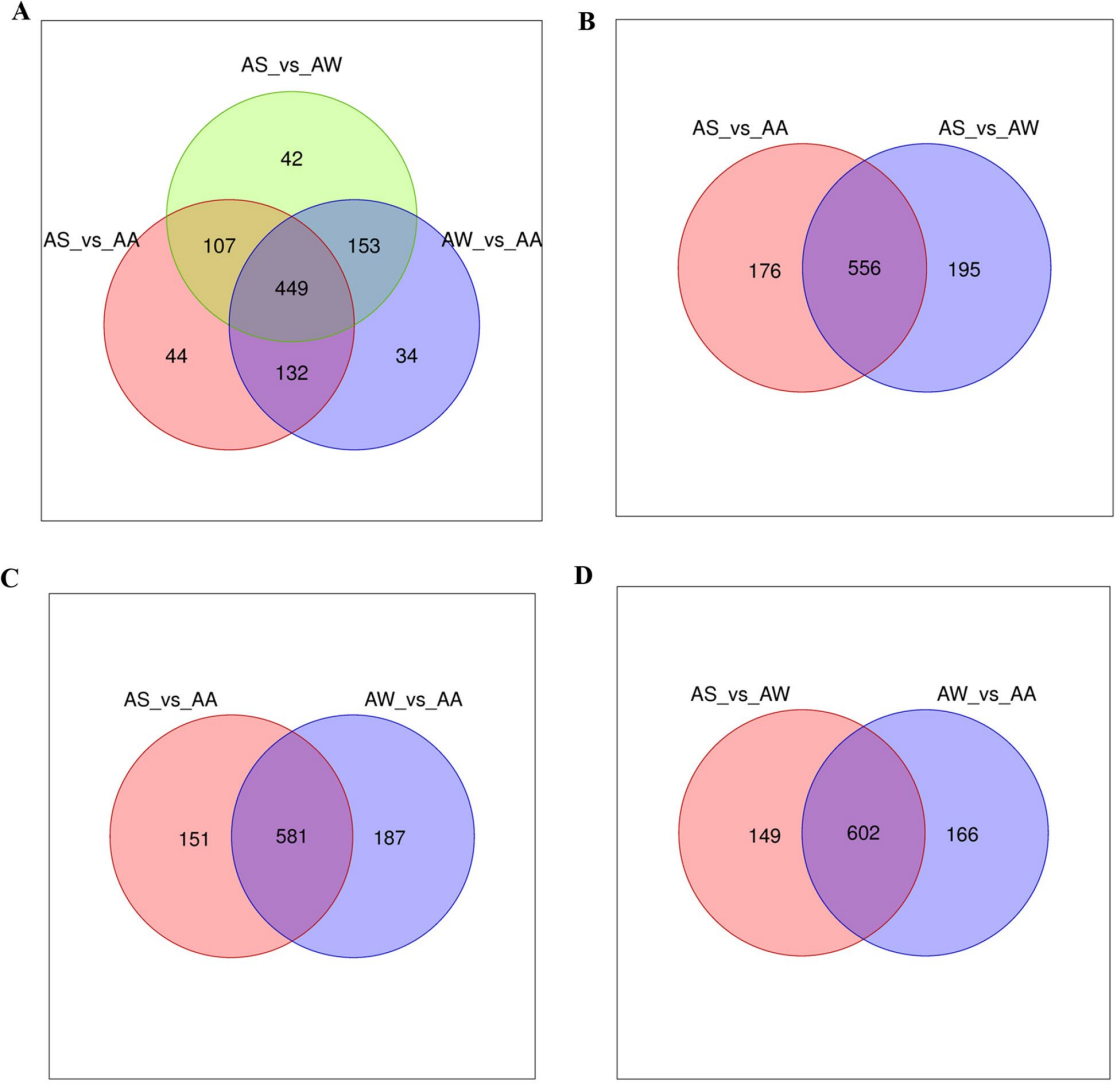
Wayne diagram of the differential metabolite. **A** All samples Wayne diagram. **B** AS vs AA Wayne diagram. **C** AS vs AW Wayne diagram. **D** AW vs AA Wayne diagram

The results of differential metabolites can be shown using Volcano and Wayn maps. Volcano plots visually demonstrated the overall distribution of different metabolites and the results are shown in Fig. 5 showing significant differences between the three *Artemisia* species. The visual display of specific metabolites and their differences were used for functional analysis of metabolic pathways, with upregulation in red, and downregulation in green and no changes in gray. There were 449 different metabolites of different species identified by the multivariate statistical analyses (Fig. 6).

### Analysis of the differential metabolites

Table 1 shows the top ranking of 20 differentially expressed metabolic components in the fold change of the distinct metabolites in *A. sieversiana* and *A. annua*. Compared with *A. annua*, clear differences could be seen in *A. sieversiana* regarding the contents of 2,6-Dimethoxybenzoic acid, Blumeatin, Luteolin-6-C-glucoside, Ethyl maltol, Luteolin-8-C-glucoside, 4,5-Epoxyartemisinic Acid, ageconyflavone B, Fraxidin and Chrysosplenetin.

**Table 1:** Significant analysis results of different metabolites (AS vs AA)

ID	name	FC	log2FC	*P* value	VIP
meta343	2,6-Dimethoxybenzoic acid	59.29223043	5.901572443	0.000453148	1.145964649
meta658	Blumeatin	52.49005062	5.714354804	0.001073898	1.144602206
meta320	4,5,6-Trihydroxy-2-oxohexanoic acid	48.08724597	5.590121722	0.000452661	1.146005986
meta887	Luteolin-6-C-glucoside (Isoorientin)	44.86251823	5.473779837	0.010383312	1.12360749
meta142	Ethyl maltol	43.70729347	5.476002669	0.000129463	1.146531467
meta885	Luteolin-8-C-glucoside (Orientin)	42.63849901	5.403909759	0.008163143	1.128568373
meta1036	Apigenin-7-O-(2″-glucosyl)arabinoside	38.57964324	5.256052698	0.014713054	1.114014763
meta512	4,5-Epoxyartemisinic Acid	37.92059039	5.244933346	0.000425748	1.146087638
meta801	ageconyflavone B	37.45372773	5.224856588	0.001870104	1.142787628
meta454	Fraxidin (8-Hydroxy-6,7-dimethoxycoumarin)	35.48432403	5.155703751	0.000687405	1.145381323
meta558	1-Octadecanol	0.947153315	-0.078149557	0.03584419	1.025840368
meta830	Chrysosplenetin (Quercetagetin-3,6,7,3'-tetramethyl ether)	0.929398985	-0.105667858	0.002185414	1.105741857
meta105	L-Isoleucine	0.907491432	-0.140079689	0.000918592	1.119349317
meta829	5,4'-Dihydroxy-3,6,7,3'-tetramethoxyflavone	0.89770689	-0.155625006	0.003404358	1.097827481
meta108	Dimethylmalonic acid	0.896490345	-0.156907437	0.041687943	1.004257483
meta831	5,7-Dihydroxy-6,3',4',5'-tetramethoxyflavone (Arteanoflavone)	0.892933182	-0.163383385	0.00036662	1.129268958
meta832	Hymenoxin	0.892933182	-0.163383385	0.00036662	1.129268958
meta1006	LysoPC 18:1	0.88961001	-0.168601589	0.007739459	1.075265358
meta106	4-Hydroxy-2-Oxopentanoic Acid	0.885009623	-0.175243707	0.044228408	1.005689678
meta487	Palmitaldehyde	0.884632045	-0.177099367	0.009399126	1.056906038

Table 2 shows the top 20 differential metabolites in the samples of *A. sieversiana* and *A. wellbyi*. Compared to *A. sieversiana*, *A. wellbyi* showed higher levels of 2-Phenylphenol, Reynosin, Rhamnetin, Methyl Cinnamate, Phenyl acetate, 4-Hydroxyacetophenone, Phloretin-4'-O-glucoside (Trilobatin), and Hispidulin-7-O-(6-O-p-Coumaroyl) Glucoside.

**Table 2:** Significant analysis results of different metabolites (AS vs AW)

ID	name	FC	log2FC	*P* value	VIP
meta457	leptodactylone	2098.044111	11.16830122	0.000658719	1.136363247
meta292	2-Phenylphenol	695.9556598	9.452316134	0.000857311	1.135926275
meta1080	Kaempferol-3-O-glucuronide-7-O-glucoside	260.7202297	8.032066997	0.000207022	1.137393473
meta509	Reynosin	180.4945683	7.590661888	0.00049123	1.136692587
meta699	Rhamnetin	171.7958993	7.428149316	0.001807675	1.133774131
meta245	Methyl Cinnamate	166.001074	7.369354404	0.005199968	1.126144979
meta128	Phenyl acetate	141.0923468	7.140225355	0.000409622	1.136936725
meta127	4-Hydroxyacetophenone	132.057566	7.046389289	0.000467111	1.136799161
meta870	Phloretin-4'-O-glucoside (Trilobatin)	116.6382363	6.872666598	0.001473688	1.134503716
meta1066	Hispidulin-7-O-(6″-O-p-Coumaroyl)Glucoside	101.3643666	6.660846975	0.00221286	1.132860912
meta518	2'-Deoxyadenosine	0.92000847	-0.120357828	0.003712148	1.085479595
meta103	L-Norleucine	0.902622552	-0.147206635	0.037241852	1.032235691
meta95	Methylenesuccinic acid	0.896792686	-0.156727072	0.021977362	1.064697458
meta30	Choline	0.889313656	-0.169740252	0.014778116	1.039332245
meta517	5'-Deoxyadenosine	0.875511585	-0.191768476	0.00033984	1.12704499
meta125	D-Threonic Acid	0.862693861	-0.213120012	0.003678779	1.082133103
meta168	L-Lysine	0.857318872	-0.220749306	0.042877212	1.043971536
meta11	2-Picoline; 2-Methylpyridine	0.845431376	-0.242313779	0.00015257	1.130152104
meta425	2,4-Di-Tert-Butylphenol	0.843832195	-0.246279225	0.017183353	1.062294717
meta571	5-Linolenic Acid	0.841495433	-0.249102924	0.002070563	1.095440959

Table 3 shows the top 20 differentially expressed metabolites in *A. wellbyi* and *A. annua*. Compared with *A. annua*, *A. wellbyi* contains more 4,5-Epoxyartemisinic Acid, Dihydro Artemisinin-D3, 2,6-Dimethoxybenzoic acid, Dihydro-epi-arteannuin B, 2-Hydroxy-3-phenylpropanoic acid, 1-O-Vanilloyl-D-Glucose.

**Table 3:** Results of significant analysis of differential metabolites (AW vs AA)

ID	name	FC	log2FC	*P* value	VIP
meta512	4,5-Epoxyartemisinic Acid	62.59060834	5.978035681	0.000396981	1.12391407
meta603	Dihydro Artemisinin-D3	58.44296782	5.868553484	0.001311209	1.121909911
meta320	4,5,6-Trihydroxy-2-oxohexanoic acid	55.48803958	5.796662565	0.000453023	1.123824104
meta343	2,6-Dimethoxybenzoic acid	50.71522251	5.664407742	0.000481078	1.123775734
meta515	Dihydro-epi-arteannuin B	49.45390688	5.626853015	0.001641491	1.12116944
meta347	2',4'-Dihydroxy-6'-methoxyacetophenone	49.19940265	5.628548044	0.000423625	1.12383703
meta267	2-Hydroxy-3-phenylpropanoic acid	43.829726	5.460712568	0.000143227	1.124460681
meta726	1-O-Vanilloyl-D-Glucose	43.20138137	5.435199222	0.000165642	1.124461282
meta541	Desacetylovatifolin	37.50735506	5.248507934	0.000965767	1.12242661
meta580	6,8-Dihydroxy-2-(2-phenylethyl)chromone	35.02656827	5.138595933	0.001278014	1.121844737
meta45	L-Proline	0.913415501	-0.13075193	0.003557826	1.075326895
meta281	3-Hydroxymandelate	0.908275906	-0.138657846	0.00665388	1.073830351
meta996	LysoPC 18:3	0.903803105	-0.146214419	0.009882893	1.054823822
meta222	2,5-Dihydroxybenzoic acid; Gentisic Acid	0.902969605	-0.147416322	0.007845477	1.045813655
meta333	D-Galactose	0.89573659	-0.159165607	0.012356376	1.028504697
meta224	3,4-Dihydroxybenzoic acid (Protocatechuic acid)	0.893907626	-0.162065364	0.008748309	1.092246215
meta262	6-Methylmercaptopurine	0.879599454	-0.185488325	0.011825769	1.088580171
meta1006	LysoPC 18:1	0.879548747	-0.185375484	0.003178125	1.099689086
meta105	L-Isoleucine	0.866973414	-0.205630476	0.007541698	1.083742832
meta335	D-Mannose	0.85013354	-0.233521474	0.017000245	1.054597848

**Fig. 7:**
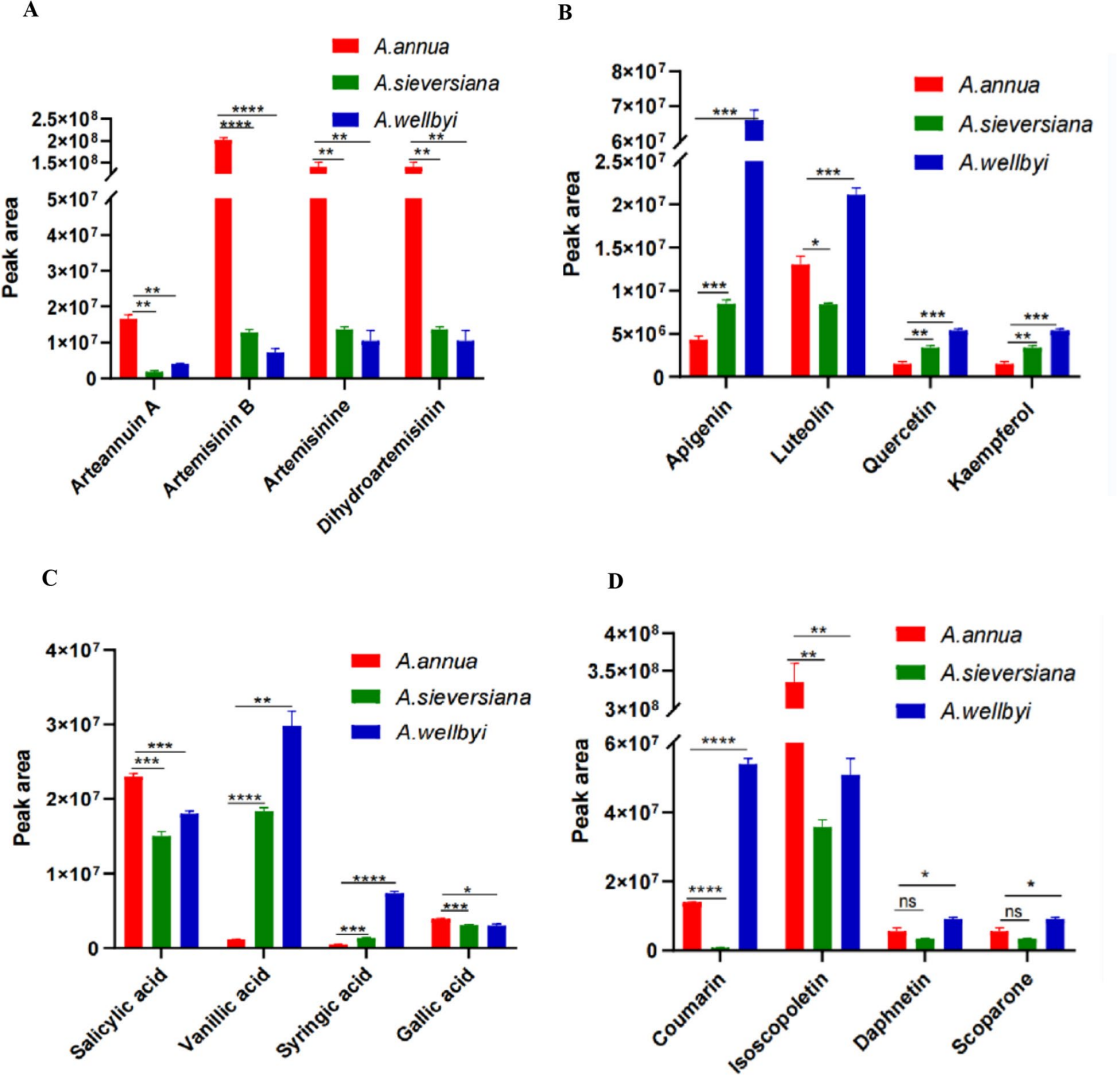
Peak area integration of the main medicinal functional metabolites of the three *Artemisia* species. **A** Artemisinin metabolites. **B** Flavonoid metabolites. **C** Phenolic acid metabolites. **D** Coumarin-class metabolites

The medicinal important metabolites from different species were assayed and compared by three plants (Fig. 7). We identified a total of 227 flavonoids from the three *Artemisia* species, accounting for 20.4% of the metabolite species. These included flavonoids such as Luteolin, Quercetin, Kaempferol, and Apigenin, which were enriched in AW. Fifty-six terpenoid metabolites were identified, including sesquiterpenoids with important pharmacological effects, such as Artemisinine, Arteannuin A, Artemisinin B, and Dihydroartemisinin. Artemisinin was found in the highest content in *A. annua*, followed by *A. sieversiana*. Coumarin, Isoscopoletin and Scoparone were in highest levels in *A. wellbyi.* Salicylic acid was in the highest level in *A. sieversian*, while Vanillic acid was found in the highest levels in *A. wellbyi*.

### KEGG enrichment analysis

**Fig. 8:**
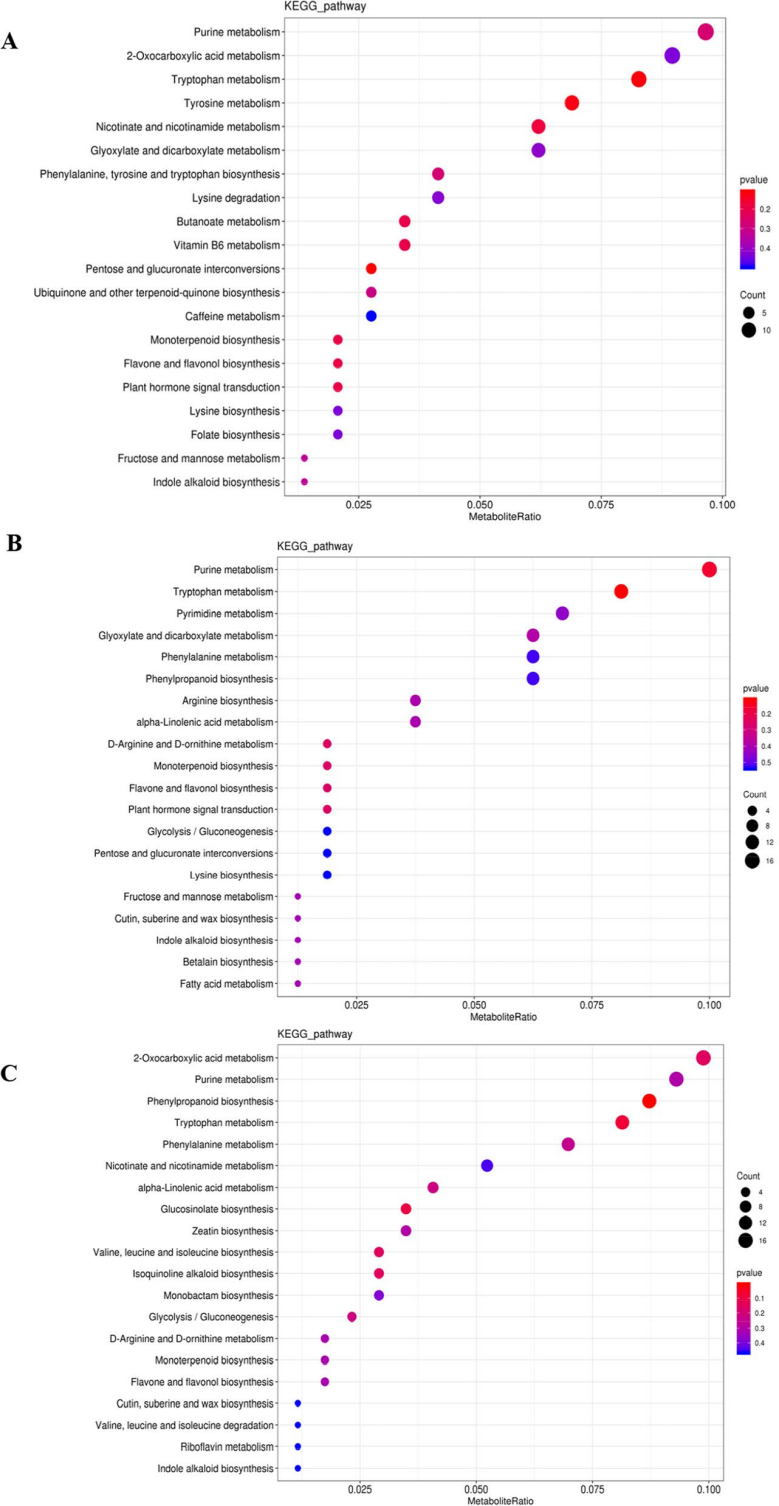
KEGG enrichment bubble chart on specific characteristics of the important metabolites. **A** AS vs AA KEGG enrichment bubble chart. **B** AS vs AW KEGG enrichment bubble chart. **C** AS vs AW KEGG enrichment bubble chart

**Fig. 9:**
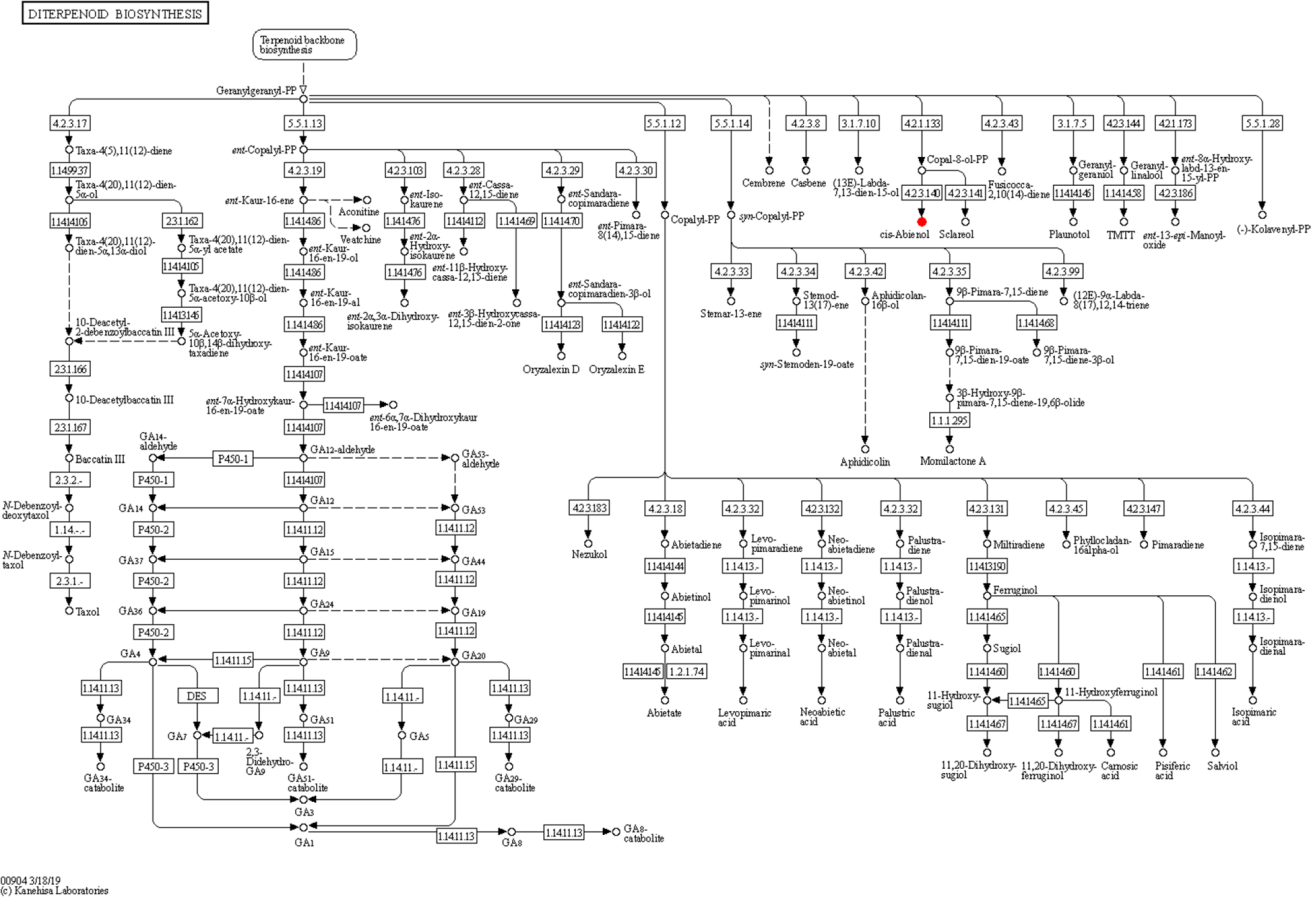
Diterpenoid Biosynthesis

The differential metabolites of AS group and AA group were mainly enriched in the Purine metabolism pathway, 2-Oxocarboxylic acid metabolism pathway, and Tryptophan metabolism pathway. The differential metabolites of AS group and AW group were mainly enriched in the Purine metabolism pathway, as well as Tryptophan metabolism pathway. The differential metabolites in AW group and AA group were mainly enriched in the 2-Oxocarboxylic acid metabolism pathway, Purine metabolism pathway, and Phenylpropanoid biosynthesis pathway. In these comparison groups, some metabolic pathways overlap, such as Purine metabolism pathway, Tryptophan metabolism pathway, 2-Oxocarboxylic acid metabolism pathway (Fig. 8). Diterpenoid Biosynthesis metabolic pathway related to differential metabolites and bioactive components (Fig. 9).

### Antibacterial activity of plant extracts

Table 4 shows that each extraction partition of *A. sieversiana* extract inhibits *E. coli*, *Salmonella*, *Streptococcus*, *S. aureus*, *P. mirabilis*, *B. cereus*, and *P. aeruginosa* differently. When the mass concentration of each extract was 200 mg/mL, Petroleum ether had better inhibitory effect on these 7 kinds of bacteria and Petroleum ether had the strongest inhibitory effect on *S. aureus* (*p* < 0.01).

**Table 4:** The inhibition zone diameters of *A. sieversiana* extract against tested bacteria (mm)

Bacteria for test	Petroleum ether layer	Chloroform layer	Ethyl acetate layer	N-butyl layer	Water layer	DMSO	Positive controls
*Bacilus cereus*	21.60 ± 0.85^***^	16.70 ± 1.18^*^	17.13 ± 1.23^*^	12.87 ± 0.45^**^	9.17 ± 0.21	-	15.72 ± 0.58
*Staphylococcus aureus*	23.83 ± 1.39^**^	21.17 ± 2.69^*^	18.07 ± 0.21^***^	13.10 ± 1.81	10.07 ± 0.12	-	20.47 ± 1.09
*Streptococcus*	19.17 ± 0.25^***^	14.10 ± 0.53^*^	17.13 ± 0.53^***^	11.47 ± 0.37	10.93 ± 0.31	-	17.53 ± 0.73
*Proteus mirabilis*	24.63 ± 1.11^**^	19.53 ± 0.76^***^	19.30 ± 0.59^***^	12.03 ± 0.25^**^	9.43 ± 0.41	-	18.30 ± 0.89
*Salmonella*	22.37 ± 0.66^***^	21.03 ± 1.43^**^	14.33 ± 0.31^***^	13.13 ± 0.87^*^	9.87 ± 0.33	-	22.43 ± 1.53
*Escherichia coli*	22.10 ± 2.36	15.70 ± 0.91^*^	20.60 ± 0.88^**^	18.30 ± 0.71^*^	9.50 ± 0.24	-	20.40 ± 0.98
*Pseudomonas aeruginosa*	22.47 ± 0.71^***^	19.00 ± 0.14^*^	20.57 ± 0.93^**^	14.43 ± 0.41	10.67 ± 0.95	-	20.70 ± 0.90

*Notes*: ^*^p < 0.05, ^**^p < 0.01, ^***^p < 0.001

Similarly, each extraction partition of the *A. wellbyi* extract has different degrees of inhibition to *E. coli*, *Salmonella*, *Streptococcus*, *S. aureus*, *P. mirabilis*, *B. cereus*, and *P. aeruginosa* (Table 5)*.* When the mass concentration of each extract was 200 mg/mL, from the perspective of the inhibition degree of each organic relative to various bacteria, the petroleum ether had better inhibitory effect on these 7 kinds of bacteria and petroleum ether had the strongest inhibitory effect on *Streptococcus* (*p* < 0.001)*.*

**Table 5:** The inhibition zone diameters of *A. wellbyi* extract against tested bacteria (mm)

Bacteria for test	Petroleum ether layer	Chloroform layer	Ethyl acetate layer	N-butyl layer	Water layer	DMSO	Positive controls
*Bacilus cereus*	26.55 ± 1.05^***^	21.72 ± 0.48^***^	11.10 ± 0.98	12.15 ± 0.72	9.90 ± 0.38	-	15.72 ± 0.58
*Staphylococcus aureus*	23.83 ± 1.39^**^	21.17 ± 2.69^*^	18.07 ± 0.21^***^	13.10 ± 1.81	10.07 ± 0.12	-	20.47 ± 1.09
*Streptococcus*	29.50 ± 0.64^***^	21.50 ± 0.41^***^	11.50 ± 0.60	11.80 ± 0.56	11.97 ± 0.49	-	17.53 ± 0.73
*Proteus mirabilis*	29.10 ± 1.21^***^	22.55 ± 0.44^***^	12.30 ± 1.03	11.90 ± 0.95	10.20 ± 0.34	-	18.30 ± 0.89
*Salmonella*	22.75 ± 1.03^***^	11.70 ± 1.39	11.15 ± 0.34	11.00 ± 0.64	11.85 ± 0.72	-	22.43 ± 1.53
*Escherichia coli*	25.23 ± 0.65^***^	21.80 ± 0.66^***^	12.70 ± 0.72	11.03 ± 0.38	12.73 ± 0.47	-	20.40 ± 0.98
*Pseudomonas aeruginosa*	24.35 ± 2.45^**^	12.50 ± 1.00	11.65 ± 0.68	10.05 ± 0.04	11.50 ± 1.01	-	20.70 ± 0.90

*Notes:* ^*^p < 0.05, ^**^p < 0.01, ^***^p < 0.001

In addition, each extraction part of *A. annua* extract has different degrees of inhibition to *E. coli*, *Salmonella*, *Streptococcus*, *S. aureus*, *P. mirabilis*, *B. cereus*, and *P. aeruginosa* (Table 6)*.* Petroleum ether had better inhibitory effect on these 7 kinds of bacteria and Petroleum ether had the strongest inhibitory effect on *P. mirabilis* (*p* < 0.001).

**Table 6:** The inhibition zone diameters of *A*. *annua* extract against tested bacteria (mm)

Bacteria for test	Petroleum ether layer	Chloroform layer	Ethyl acetate layer	N-butyl layer	Water layer	DMSO	Positive controls
*Bacilus cereus*	26.55 ± 2.05^**^	9.72 ± 0.46	13.10 ± 1.98	12.15 ± 0.92	9.30 ± 0.28	-	15.72 ± 0.58
*Staphylococcus aureus*	24.65 ± 2.23^**^	11.15 ± 0.50	10.65 ± 0.21	10.95 ± 0.07	11.55 ± 1.91	-	20.47 ± 1.09
*Streptococcus*	20.70 ± 0.74^**^	9.17 ± 0.31	10.50 ± 0.70	8.80 ± 0.36	10.57 ± 0.59	-	17.53 ± 0.73
*Proteus mirabilis*	31.00 ± 1.41^***^	10.55 ± 0.64	11.30 ± 1.13	10.90 ± 0.85	9.20 ± 0.14	-	18.30 ± 0.89
*Salmonella*	21.75 ± 1.06^***^	11.90 ± 3.39	11.25 ± 0.64	11.30 ± 1.84	9.85 ± 0.92	-	22.43 ± 1.53
*Escherichia coli*	23.23 ± 0.75^***^	8.80 ± 0.36	10.70 ± 0.72	9.83 ± 0.35	9.73 ± 0.57	-	20.40 ± 0.98
*Pseudomonas aeruginosa*	25.35 ± 4.45^*^	11.00 ± 0.00	11.95 ± 0.64	11.65 ± 0.92	10.80 ± 1.84	-	20.70 ± 0.90

*Notes:* ^*^p < 0.05, ^**^p < 0.01, ^***^p < 0.001

### Antioxidant activity of plant extracts

**Fig. 10:**
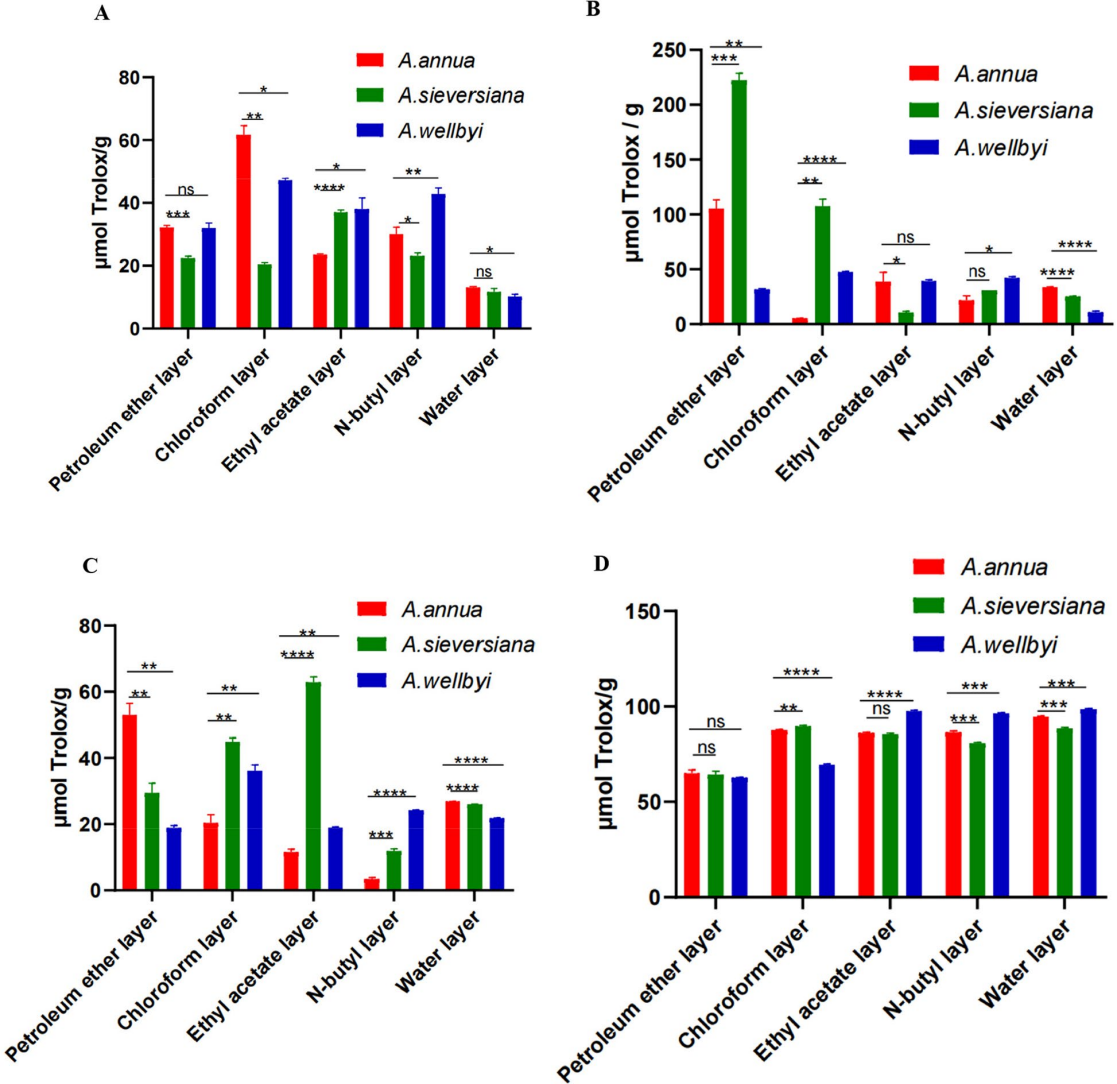
Antioxidant activity of the three *Artemisia* species. **A** FRAP assay. **B** Scavenging capacity of DPPH free radicals. **C** Scavenging activity against ABTS free radical. **D** Determination of hydroxyl radical scavenging capacity

The ethyl acetate extraction of *A. sieversiana* plant showed the strongest antioxidant capacity, followed by n-butanol extraction and petroleum ether extraction. In contrast, the dichloromethane extraction of *A. annua* plant had the strongest antioxidant capacity, followed by petroleum ether partition and n-butanol partition. The dichloromethane extraction of *A. wellbyi* had the strongest antioxidant capacity, followed by the n-butanol extraction and the ethyl acetate extraction. The water extracts of the three plants had the weakest antioxidant capacity. Among the three species, the dichloromethane extraction of *A. annua* has the strongest antioxidant capacity (Fig. 10A).

DPPH is a stable free radical, soluble in polar solvents such as methanol and ethanol, and has a large absorption at 515 nm. When antioxidants are added to the DPPH solution, a decolorization reaction occurs, so the change in absorbance can be used to quantify the antioxidant capacity of antioxidants with Trolox as a control system. The petroleum ether part of *A. sieversiana* had the strongest scavenging ability to DPPH free radicals, followed by methylene chloride and n-butanol, and the ethyl acetate part had the weakest scavenging ability to DPPH free radicals; The petroleum ether part of *A. annua* plant had the strongest scavenging ability to DPPH free radical, followed by ethyl acetate part, and the dichloromethane part has the weakest scavenging ability to DPPH free radical; The scavenging ability of DPPH free radical was the strongest in the dichloromethane part of *A. wellbyi* followed by the n-butanol part and the ethyl acetate part, and the water extract had the weakest scavenging ability on DPPH free radical. Among the three species, the petroleum ether part of *A. sieversiana* has the strongest scavenging ability to DPPH free radicals (Fig. 10B).

The dichloromethane extraction of *A. sieversiana* plant had the strongest scavenging ability to ABTS free radical, followed by petroleum ether partition, and the ethyl acetate part had the weakest scavenging ability to ABTS free radical. The petroleum ether part of *A. annua* plant had the strongest scavenging ability to ABTS free radicals, followed by the methylene chloride part, and the n-butanol part had the weakest scavenging ability to ABTS free radicals. The ethyl acetate part of *A. wellbyi* plant had the strongest scavenging ability to ABTS free radical, followed by n-butanol part, and the petroleum ether part had the weakest scavenging ability to ABTS free radical. Among the three species, the dichloromethane site of *A. sieversiana* had the strongest scavenging ability to ABST free radicals (Fig. 10C).

H_2_O_2_/Fe^2+^ generates hydroxyl radicals through the Fenton reaction, and salicylic acid can effectively capture the generated hydroxyl radicals and react with them to form a colored substance, 2,3-dihydroxybenzoic acid. After the substance is removed, the colored substances will be reduced, so that the ability of the sample to scavenge hydroxyl radicals can be judged according to the value of the absorbance value. The dichloromethane part of *A. sieversiana* plant had the strongest scavenging ability to hydroxyl radicals, followed by water extract, and the petroleum ether part has the weakest scavenging capacity to hydroxyl radicals; The water extract of *A. annua* plant had the strongest scavenging ability to hydroxyl free radicals, followed by the dichloromethane part, and the petroleum ether part had the weakest scavenging ability; The water extract of *A. wellbyi* has the strongest scavenging ability, followed by ethyl acetate, and petroleum ether had the weakest scavenging ability. Among the three plants, the water extract of *A. serrata* had the strongest scavenging ability (Fig. 10D).

## Discussion

In this study, we used widely targeted metabolomics to analyze the primary and secondary metabolites of three *Artemisia* species collected from Tibet, and identified 1109 metabolites in 10 categories. This compares to the total of 535 metabolites identified using non-targeted metabolomics to analyze three species of *Artemisia* [24]. The main metabolites identified here were flavonoids, phenolic acids, lipids, amino acids and their derivatives, organic acids, alkaloids, and terpenes. The important pharmacologically active compounds are flavonoids, phenolic acids, artemisinins and coumarin compounds.

The metabolites of three *Artemisia* species were identified by widely-targeted metabolomics technology, and a total of 227 flavonoids were obtained. Flavonoids were the most abundant metabolites, accounting for 20.4% of the total metabolites. Flavonoids are widely present in *Artemisia* and are an important class of natural organic compounds [25]. Zhang [26] et al. identified 10 flavonoids from *A. sphaerocephala*. Among them, the representative quercetin has a wide range of pharmacological effects in antioxidant, anti-inflammatory and antibacterial, anti-tumor [27–29]. In addition to these documented compounds, we also detected 42 sesquiterpenoids from these 3 species of *Artemisia*, such as artemisinin, artemisinin A, artemisinin B, artemisinic acid, dihydroartemisinic acid, etc. Previous studies have found that artemisinin compounds such as ATS can inhibit βIL-1, IL-6, IL-17α and other inflammatory cytokines, suggesting that they play the roles of anti-inflammatory, anti-angiogenesis, inhibiting autoimmune arthritis and treating rheumatoid arthritis [30]. Phenolic acids have significant effects in anti-inflammatory, anti-allergic, vascular protection, antioxidant activity, anti-tumor, anti-bacterial and fungal and liver protection [31] Coumarin compounds have good physiological and pharmacological activities in antiviral, antifungal, anti-tumor, and anti-inflammatory aspects [32].

The antibacterial experiments of the three *A.* species showed that the different polar solvent extracts from the three *A.* species had strong antibacterial activities. Zohra [33] et al. used an aqueous extract of *A. annua* against 3 Gram-negative bacteria and 3 Gram-positive bacteria were evaluated for bacteriostatic activity. Although the antibacterial activity of ACAE is lower than that of ampicillin, at a concentration of only 50 mg/mL, it has the strongest inhibition zone (13 mm) with good inhibition against *S. aureus.* The study by Darwish [34] et al. showed that the methanol extract of this plant has high antibacterial activity. Widely targeted metabolomics results revealed the presence of derivatives such as flavonoids, terpenoids, phenols and alkaloids. In addition, alkaloids, flavonoids, phenols, and terpenes in various plant extracts have all been shown to be effective antibiotics. Our results are also consistent with these studies showing that these 3 *Artemisia* species have efficacy against clinical pathogens.

The antioxidant activity test showed that the three species of *Artemisia* have strong antioxidant capacity in vitro. The antioxidant activity experiments of *A. sieversiana* essential oil by Li [35] et al. showed that the IC_50_ of *A. annua* essential oil on DPPH free radicals, ABTS^+^ and hydroxyl radicals were lower than vitamin _C_, indicating that *A. sieversiana* essential oil had strong in vitro antioxidant capacity, which is stronger than V_C_. This flavonoid purified product has scavenging ability for hydroxyl radicals, antioxidant activity to grease, that is stronger than citric acid. It has stronger antioxidant activity on vegetable oils than ascorbic acid, and slightly weaker than ascorbic acid on animal fats and oils. The residue of *A. annua* is rich in flavonoids and has strong antioxidant activity, which is a natural antioxidant. Our findings are consistent with some studies [36] showing that 3 *Artemisia* species have antioxidant effects, and their antioxidant properties may be related to the phenolic and flavonoid content of *Artemisia*.

We found that *A. sieversiana* and *A. wellbyi* collected from Tibet, are likely to have the same antibacterial and antitumor properties as widely reported *A. annua.* It has great potential medicinal value in pharmacological effects such as antiviral and anti-inflammatory [37, 38], therefore, we have reason to believe that *Artemisia* sp. have an extensively application prospect on medicine and feed additives in Tibet in future.

## Conclusions

This study identified and quantified the metabolites from three *Artemisia* species collected from Tibet using widely targeted metabolomics technology. The types of screened and identified differential metabolites were mainly flavonoids, phenolic acids, artemisinins and coumarins. The antibacterial experiments showed that the three *Artemisia* species had strong antibacterial activities against *B. subtilis*,* E. coli*, *S. aureus*, *P. mirabilis* and *P. aeruginosa*. The antioxidant activity test showed that the three species of *Artemisia* have strong antioxidant capacity in vitro, these wide ranges of beneficial effects suggest great potential for these components for future therapeutic applications.

## Materials and methods

### Plant materials

Samples of *A. sieversiana*, *A. wellbyi* and *A. annua* were collected from Jinbei, Caina Township, Qushui County, Lhasa City, Tibet Autonomous Region in July 2020 (east longitude 90°53′ 58.60", north latitude 29°26′ 6.03", elevation 3581 m). Official permits for collection of these native plants were not required because these plants are not included in the list of national key protected plants, however permission for collections was obtained from the Lhasa Forestry and Grassland Administration. The formal identification of the plant material was performed by Professor Zhaoyang Chang of College of Life Science, Northwest A&F University based on morphological characters. The specimens of *A. sieversiana*, *A. wellbyi*, and *A. annua* have been deposited at Herbarium, Institute of Botany, Chinese Academy of Sciences (voucher # PE01890226, PE01890481, PE01997408, respectively). Sample collection of Plants were from each 10 m × 10 m sampling site; 3 plants were collected diagonally with a total of 9 plants/site. All samples were dried, crushed, passed through a 40-mesh sieve (with an aperture of 0.425 mm), put into a paper bag, and stored in a desiccator at room temperature for later use. One g each of 9 samples were wrapped in tin foil, snap frozen in liquid nitrogen for storage, transported in dry ice to Biomarker Technology Co., Ltd. for analysis.

### Chemical reagents and instruments

Methanol (Merck, Germany), acetonitrile (Merck, Germany), formic acid (Merck, Germany), pipette (Thermo company, USA), freeze dryer (Scientz company, Germany), grinder (Retsch company, Germany), UPLC (SHIMADZU, Japan), Tandem mass spectrometry (ABI, USA), column (Agilent, Germany), industrial alcohol, petroleum ether, ethyl acetate, dichloromethane, n-butanol, dimethyl sulfoxide, peptone, beef extract, agar powder, sodium chloride, etc. Pressure steam sterilizer (Shanghai Boxun), electronic balance PTX-FA110 (Sartorius, Germany), ultra-clean workbench (Suzhou purification), refrigerator (FRESTECH SC-208A), water bath, microwave oven (Foshan Midea), rotary steamer.

### Widely targeted metabolomics experiment

#### Metabolite extraction

The main processing steps are as follows: the biological samples are freeze-dried in vacuum (Scientz-100F), and the dried samples are ground in a grinder (MM 400, Retsch) at 30 Hz for 1.5 min to powder. Dissolve 100 mg of powder sample in 1.2 mL of 70% methanol, mix it with vortex every 30 min for 30 s each time for 6 times, and store it at 4 °C overnight. The sample was centrifuged at 12,000 rpm for 10 min. Suck the supernatant through the hole diameter of 0.22 μ M and stored for UPLC-MS/MS analysis.

The LC/MS system for metabolomics analysis is composed of Waters Acquity I-Class PLUS ultra-high performance liquid tandem Waters Xevo G2-XS QT of high resolution mass spectrometer. The column used is purchased from Waters Acquity UPLC HSS T3 column (1.8 μm 2.1*100 mm). Positive ion mode: mobile phase A: 0.1% formic acid aqueous solution; mobile phase B: 0.1% formic acid acetonitrile. Negative ion mode: mobile phase A: 0.1% formic acid aqueous solution; mobile phase B: 0.1% formic acid acetonitrile. Injection volume 1μL.

Waters Xevo G2-XS QTOF high resolution mass spectrometer can collect primary and secondary mass spectrometry data in MSe mode under the control of the acquisition software (MassLynx V4.2, Waters). In each data acquisition cycle, dual-channel data acquisition can be performed on both low collision energy and high collision energy at the same time. The low collision energy is 2 V, the high collision energy range is 10-40 V, and the scanning frequency is 0.2 s for a mass spectrum. The parameters of the ESI ion source are as follows: Capillary voltage: 2000 V (positive ion mode) or -1500 V (negative ion mode); cone voltage: 30 V; ion source temperature: 150 °C; desolvent gas temperature 500 °C; backflush gas flow rate: 50L/h; Desolventizing gas flow rate: 800L/h.

### Antibacterial assays

#### Extraction of active components from plants

The dried plant samples (100 ɡ) were ground, soaked in industrial alcohol, extracted under reflux at 60 °C for 3 h, concentrated by rotary evaporation under reduced pressure in water bath held at 55 °C. The total ethanol extracts were obtained by heating and drying for 12 h. Thirty g of the ethanol extract was dissolved in 800 mL water, and was extracted in 1-L flask with petroleum ether, chloroform, ethyl acetate and n-butanol. The extracts were condensed under vacuum and dried at 55 °C. The extracts of each extraction were dissolved in dimethyl sulfoxide separately, prepared into a solution in a concentration of 200 mg/mL, filtered through a 0.22 μm filter, and stored at 4 °C for later use.

#### Medium preparation

Nutrient agar medium (1000 ml containing 3.0 g beef extract, 10.0 ɡ peptone, NaCl 5.0 ɡ and 20 ɡ agar (pH 7.2–7.4).

#### Bacterial liquid preparation

*E. coli*, *Salmonella* (G^−^), *P. mirabilis* (G^−^), *B. cereus* (G^+^), *S. aureus* (G^+^), *Streptococcus* (G^+^), *P. aeruginosa*, (G^−^). The above strains were provided by the Microbiology Laboratory of Northwest A&F University. Under aseptic conditions, single colonies of activated *E. coli*, *Salmonella*, *Streptococcus*, *S. aureus*, *P. mirabilis*, *B. cereus*, and *P. aeruginosa* after activation were picked with an inoculation loop and inoculated into the liquid medium, respectively, at a constant temperature of 37 °C. Cultivated for 24 h. Use sterilized liquid medium to adjust the concentration of each bacterial solution equivalent to 0.5 McFarland turbidity standard (about 1.5 × 10^8^ CFU/mL) for use.

#### Agar well diffusion assay

A sterilized filter paper sheet, 6 mm dia. was soaked in a drug extract for 1 h. The bacterial suspension to be tested (0.5 mL) was spread evenly onto the surface of the solid medium. The filter paper of the extract was layered onto the bacteria-containing plate under sterile conditions; a sterile filter paper soaked in DMSO was used as the negative control, and the ceftazidime drug sensitive tablet was used as the positive control. The leaching solution treatment and control were incubated (37 °C, 12 h); inhibition zone of the filter paper was measured. The experiment was repeated 3 times. If the diameter of the inhibition zone is greater than or equal to 18 mm, the bacteria is rated as highly sensitive, 12 to 18 mm as moderately sensitive, 7 to 12 mm as low sensitivity, and less than 7 mm as insensitive.

### Determination of antioxidant activity

#### FRAP assay

Sample (1 ml) was mixed with 2.5 mL of phosphate buffer (0.2 moL/L, pH 6.6) and 2.5 mL of 1% K_3_Fe(CN)_6_, and heated in a water bath (50 °C, 20 min). After incubation, 2.5 mL of 10% (w/v) trichloroacetic acid was added and the samples were centrifuged (15,000 × ɡ, 10 min) and 2.5 mL of the supernatant was aspirated and mixed with 2.5 mL of H_2_O and 0.5 mL of 0.1% FeCl_3_. The absorbance was measured with a spectrophotometer (700 nm).

#### Scavenging capacity of DPPH free radicals

To 2.0 mL of each solution to be tested, 2 mL 0.04 mg/mL of DPPH solution (with ethanol as a solvent) is added, mixed.

#### Scavenging activity against ABTS free radical

The ABTS working solution was carefully transferred into the first reagent tube, the tube cover was rotated, shaken well, and placed at temperature for 14–16 h. Take 10 μL ABTS and dilute the diluent (first 20 diluted in pure water) and record the dilution ratio. Fully mix, microabsorption values were measured at 734 nm.

#### Determination of hydroxyl radical scavenging capacity

Two mL of the samples to be tested was mixed with 1.4 mL 6 mmol/L H_2_O_2_, then 0.6 mL 20 mmol/L sodium salicylate and 2 mL 1.5 mmol/L ferrous sulfate added. Samples were thoroughly mixed; for the blank group, the sample was replaced with ultrapure water. The absorbance value of H_2_O_2_ was measured in the same way and recorded as A background. OH free radicals. The formula for calculating the clearance rate = [A blank—(A sample—A background)] / A blank × 100%.

### Data analysis

After identification of distinct compounds and pathway analysis using KEGG database, *t*-test was used to calculate the difference significance *p*-value of each compound. R package was used for the OPLS-DA modeling was performed using R package and the reliability of the model was tested with 200 times permutation.

## Supplementary Information

**Additional file 1: Supplementary 1.** Information on all metabolites in three Artemisia species.

## Data Availability

The datasets used and/or analyzed during the current study are available from the corresponding author on reasonable request.

## Abbreviations

AS: *Artemisia* *sieversiana*
AW: *Artemisia* *wellbyi*
AA: *Artemisia* *annua*
UPLC: Ultra Performance Liquid Chromatography
MS/MS: Tandem mass spectrometry
MRM: Multiple reaction monitoring
PCA: Principal component analysis
KEGG: Kyoto Encyclopedia of Genes and Genomes
PC1: First principal component
OPLS-DA: Orthogonal projections to latent structures-discriminant analysis
VIP: Variable importance in projection
DPPH: 2,2-Diphenyl-1picrylhydrazyl
FRAP: Ferric reducing antioxidant power
